# Ultrasound-Assisted Synthesis of Potentially Food-Grade Nano-Zinc Oxide in Ionic Liquids: A Safe, Green, Efficient Approach and Its Acoustics Mechanism

**DOI:** 10.3390/foods11111656

**Published:** 2022-06-04

**Authors:** Lei Zhang, Yang Hu, Xue Wang, Ao Zhang, Xianli Gao, Abu El-Gasim A. Yagoub, Haile Ma, Cunshan Zhou

**Affiliations:** 1 School of Food and Biological Engineering, Jiangsu University, Zhenjiang 212013, China; zhangleifd@ujs.edu.cn (L.Z.); hyhyhy9797@163.com (Y.H.); wxue7777@163.com (X.W.); 2222118029@stmail.ujs.edu.cn (A.Z.); gaoxianli@ujs.edu.cn (X.G.); mhl@ujs.edu.cn (H.M.); 2 Faculty of Agriculture, University of Zalingei, P.O. Box 06, Zalingei 79371, Sudan; gaschem2000@yahoo.com

**Keywords:** ultrasonic cavitation, ultrasonic mechanism, nano-zinc oxide, characterization

## Abstract

In food application, nano-zinc oxide (nano-ZnO) is a very important nano metal material; thus, it is necessary to prepare potentially food-grade nano-ZnO. Nano-ZnO synthesized by the ultrasound-assisted method can reach a safe level because of its import physical processing characteristics. Firstly, the micromorphology and microstructure of nano-ZnO synthesized by the ultrasonic method were compared with that by the mechanical stirring method through atomic force microscopy, X-ray diffraction, and Fourier-transform infrared. Secondly, the on-line monitoring of different ultrasonic fields in real-time was studied during the whole synthesis process of nano-ZnO by polyvinylidene fluoride sensor, and two control groups (water medium) were set. The results showed that nano-ZnO obtained by the ultrasonic method were smaller in size and had less surface roughness compared with the mechanical stirring method. The nucleation and crystallization process of nano-ZnO was controlled by the ultrasonic method with sharp diffraction peaks of higher intensities. Moreover, for the ultrasonic mechanism, it was found that the oscillation behavior of bubbles varied from liquid to liquid, and variation was also found in the same liquid under different restraint of interfaces. Based on voltage waveforms monitored in the three liquid media, differences in the life cycle of cavitation bubble oscillation, cycle of collapse stage, maximum voltage amplitude, and acoustic intensity were observed. The physical mechanism of ultrasound-assisted synthesis of nano-ZnO was revealed through voltage fluctuations of the acoustics signal, which can lay a theoretical foundation for the controllability of food ultrasonic physical processing.

## 1. Introduction

In food research, nano-zinc oxide (nano-ZnO), with a specific surface area bigger than conventional ZnO, is considered as a new-style functional material, and the size of nano-ZnO is classified between clusters and macroscopic particles [1]. It was reported that its characteristic of a large surface-to-volume ratio can increase the interaction with the target substance, including hydronium/hydroxyl ions in water solution [2]. Moreover, due to other advantageous characteristics, such as high adsorption capacity, non-toxicity, biocompatibility, low cost, high exciton binding energy (60 eV), excellent electron mobility, and high thermal and chemical stability, nano-ZnO has a wide range of applications, such as catalysts, sensors, photovoltaic, luminescent, electrical, and acoustic devices, coatings, micro lasers, memory arrays, and biomedical applications [2,3,4,5]. In food areas, nano-ZnO has two main effects; on the one hand, it can be used as a food packaging material with certain antibacterial and stability, and on the other hand, it can be used as a food additive to supplement the essential trace elements of the human body [6]. For example, nano-ZnO are reported as efficient antibacterial agents and wound dressing applications [7]. The photocatalytic properties of ZnO enable the absorption of ultraviolet light and the prevention of the photosensitive oxidation of fresh food, which inhibits the synthesis of mycotoxins and the proliferation of *Aspergillus niger* [8]. Therefore, nano-ZnO applications in food packaging can act as an antibacterial material; improve the performance of packaging materials, such as mechanical strength, barrier performance, and stability; and prolong the shelf-life of food [9]. Emamifar et al. [10] produced low-density polyethylene (LDPE) nanocomposite films using silver and ZnO as active packaging to extend the shelf-life of fresh orange juice by up to 28 days, with no negative effects on sensorial parameters. ZnO with different morphologies (particle size and shape) has different degrees of antibacterial activity [11]. The uniform and fine nano-ZnO, with higher surface energy and larger specific surface area, can be better dispersed in the matrix, which promotes the interface bonding between the nanoparticles and the polymer, and owns a wide antibacterial spectrum and good biocompatibility [6,12]. In addition, Zn is an essential trace element in the human body, and plays an important role in the formation of various functional enzymes in human body. According to the provisions of the Food and Drug Administration (FDA), nano-ZnO, as a recognized safety material, can be used in food additives to supplement food nutrition [13]. This is mainly because nano-ZnO has high specific surface area and unique physicochemical properties. Compared with other Zn sources, nano-ZnO has many advantages, such as high absorption rate, good biological activity, and oxidation resistance [6].

Up to now, various methods have been employed to synthesize nano-ZnO, including wet-chemical approach [14], sol-gel process [15], thermal evaporation [16], etc. Ultrasonic technology, as a new and promising technology, has been widely used for its green and efficient characteristics [17], and can effectively control the particle size and morphology of nano-ZnO for its application in food areas, such as food packaging film [18]. It takes place upon the application of high-energy ultrasound to the reaction mixture, integrating physical acoustics with chemistry. Ultrasound technology has emerged as a promising technique that is efficient, simple, and economically cost-effective in food physical processing areas [19]. The chemical effect of ultrasound arises from acoustic cavitation, which is the formation, growth, and implosive collapse of bubbles in liquid, generating local hot spots of high temperature, and high pressure [20,21]. The ultrasonic cavitation can drive a variety of reactions to synthesize nano-structure materials [22]. As a result, this method is considered as a simple, cost effective, and environmentally friendly approach to nano-structure materials, and, therefore, is anticipated to be a promising route to generate novel nano-structure materials, including nano-ZnO, under ambient conditions [20]. Ionic liquids (ILs), which entirely consist of cations and anions, are a kind of salt that are liquids under room temperature. ILs, as a new solvent system, are increasingly arousing worldwide interest due to their high mobility, thermal stability, low melting points, non-flammability, negligible vapor pressure, large electrochemical window, excellent catalytic activity, reusability, low toxicity, and ability to dissolve a variety of chemicals [23]. In this context, the synthesis of nano-structure materials in ILs assisted by the ultrasonic waves could result in an efficient, green, and good-quality nanoscale compound.

Recently, studies illustrating the ultrasound-assisted synthesis of nano-ZnO in ILs have been evolving [24,25,26,27,28,29]. The effect of ultrasonic waves on the synthesis of nano-ZnO has been interpreted based on the indirect chemical reaction behavior or analog simulation, which provides a very important theoretical basis for the development of ultrasonic applications. However, there is another question of how the ultrasonic field works itself, so the direct physical mechanism of ultrasonic cavitation needs to be studied during the ultrasound-assisted synthesis of nano-ZnO. The mechanical waves of different ultrasonic frequencies are different, resulting in different number, size, and oscillation period of cavitation bubbles, which will affect the intensity of the ultrasonic field. High-frequency ultrasonic bubbles have a narrower range of activity, shorter growth period, smaller expandable size, and less energy generated when the bubbles break [30]. In addition, liquid medium also affects the intensity of the ultrasonic field. For example, in high-viscosity liquids, the negative pressure generated in the sonic expansion stage cannot easily overcome the liquid environmental pressure, the cavitation activity is not easy to start, and the ultrasonic effect is not good [31]. By utilizing the advantages of PVDF (polyvinylidene fluoride) with high thermal stability, chemical resistance, flexibility, and high electrical activity, the mechanical energy generated when the cavitation ruptures can be converted into electrical energy and transmitted to an oscilloscope to characterize the ultrasonic field strength [32,33]. Hence, in the present research, the synthesis of nano-ZnO aided by the ultrasound waves was explained from the point of physical theory on the oscillation behavior of cavitation bubbles. This investigation might help in a future controlling of an ultrasound-assisted synthesis of a purpose-built nano-structure material.

In short, in order to improve the application of nano-ZnO in food areas, ultrasound-assisted synthesis was utilized to obtain nano-ZnO with homogeneous dispersion and stability, and the micromorphology and microstructure of nano-ZnO were analyzed by atomic force microscopy (AFM), X-ray diffraction (XRD), and Fourier-transform infrared (FTIR), as compared with that by the mechanical stirring method. Furthermore, the acoustics signals from the ultrasonic cavitation fields were evaluated based on the oscillation behavior of cavitation bubbles during the synthesis of potentially food-grade nano-ZnO to explain the mechanism of ultrasound-assisted synthesis.

## 2. Materials and Methods

### 2.1. Experimental Materials and Apparatus

Zinc acetate dihydrate (Zn (CH_3_COO)_2_·2H_2_O, AR), sodium hydroxide (NaOH, AR), 1-butyl-3-methylimidazolium tetrafluoroborate ([Bmim][BF_4_], with purity > 99%, AR), ethyl alcohol (C_2_H_5_OH, AR), water (H_2_O), and potassium bromide powder (KBr, SP) were purchased from Sinopharm Chemical Reagent Co., Ltd. (Shanghai, China).

The following equipment was used in the experiment: electronic balance (PRACTUM2102-1CN, Sartorius AG, Gottingen, Germany), magnetic stirring apparatus (MS-06SU, Crystal Technology & Industries, Inc., Dallas, TX, USA), ultrasonic processing system (KQ3200E, Kunshan Ultrasonic Instruments Co., Ltd., Suzhou, China), centrifuge (DL-5C, Shanghai Anting Scientific Instrument Factory, Shanghai, China), drying oven (BGG-9030, Shanghai Yiheng Technical Co., Ltd., Shanghai, China), atomic force microscope (AFM) (Multimode 8, Bruker Corporation, Karlsruhe, Germany), X-ray diffractometer (XRD) (D/MAX-RA(Cu Ka), Rigaku Corporation, Tokyo, Japan), powder compressing machine (FW-4A, Tianjin Science and Technology Co., Ltd., Tianjin, China), Fourier-transform infrared spectrometer (FTIR) (WQF-510A, Beijing Beifen-Ruili Analytical Instrument (Group) Co., Ltd., Beijing, China), and digital oscilloscope with four channels (GDS-3254, Good Wilzl Instrument Co., Ltd., Taiwan, China).

### 2.2. Synthesis of Nano-ZnO by Mechanical Stirring Method

In this method, nano-ZnO was synthesized by dissolving 0.4 g of Zn (CH_3_COO)_2_·2H_2_O and 0.2 g of NaOH in 40 mL of the ionic liquid, [Bmim][BF_4_], in a 100 mL beaker. Next, the reaction mixture was stirred at 450 rpm for 10 min using a magnetic stirrer. At the end of the reaction, the mixture was centrifuged at 4000 rpm for 15 min, a supernatant removed, and a white precipitate was obtained. The white precipitate was subsequently washed with ethyl alcohol and deionized water, and dried at 70 °C for 10 h. The final white powder was kept for further analysis.

### 2.3. Synthesis of Nano-ZnO by Ultrasonic Method

To synthesize nano-ZnO by the ultrasonic method, 0.4 g of Zn (CH_3_COO)_2_·2H_2_O and 0.2 g of NaOH were dissolved in 40 mL of [Bmim][BF_4_] in a 100 mL beaker. Next, the beaker was immersed into the water in the ultrasonic bath device; the total volume of water in the bath was 2300 mL. Sonication was performed at a frequency of 40 kHz with a constant output power of 150 W for 10 min, and the ultrasonic power density was 65.2 W/L. After sonication, the reaction mixture was centrifuged as before, a supernatant was removed, and a white precipitate was obtained. The white precipitate was subsequently washed with ethyl alcohol and deionized water, and dried at 70 °C for 10 h. The final white powder was kept for further analysis.

### 2.4. Determination of Nano-ZnO Micromorphology by Atomic Force Microscopy (AFM)

The micromorphology analysis of nano-ZnO on mica was performed by a multimode atomic force microscope. The nano-ZnO, synthesized by mechanical stirring and ultrasonic methods, was dissolved in ethyl alcohol to prepare a suspension with a concentration of 300 μg/mL. A 5 μL of sample suspension was deposited onto freshly-cleaved mica and dried in a hood at room temperature (25 °C). Images were scanned in the air using standard peak force-mode and a silicon cantilever. The images were analyzed using the software, Nanoscope Analysis V1.5. The surface roughness and particle size of nano-ZnO were measured. Two important indexes of surface roughness, viz, mean roughness (*R_a_*), and root-mean-square roughness (*R_q_*) were defined by using Equations (1) and (2) [34,35]:

(1) $$R_{\alpha }=\frac{1}{N}\overset{N}{\underset{i=1}{\sum }}\left|Z_{i}-\bar{Z}\right|$$

(2) $$R_{q}=\sqrt{\frac{\sum_{i=1}^{N}{\left(Z_{i}-\bar{Z}\right)}^{2}}{N}}$$

where *Z_i_* is the height of the *i*th point with regard to the lowest one, $\bar{Z}$ is the mean value of height, and *N* is the total number of points comprising the image.

### 2.5. Determination of Nano-ZnO Structure by X-ray Diffraction (XRD)

The crystal structures of nano-ZnO were investigated by the X-ray powder diffraction pattern. A wide XRD with Cu Kα radiation (*λ* = 0.1542 nm) was used. The XRD data were collected in the continuous scan mode from 15° to 80° (2*θ*), and at a scanning speed of 0.02°/min.

### 2.6. Determination of Functional Groups of Nano-ZnO by FOURIER Transform Infrared (FTIR) Spectroscopy

The infrared spectra of nano-ZnO were recorded in absorbance mode from 400 to 4000 cm^−1^ on an FTIR spectrophotometer equipped with a potassium bromide (KBr) beam splitter at 25 °C. Samples were prepared for analysis by grinding 1 mg of nano-ZnO with 100 mg of KBr, and then pressed into translucent pellets. Scanning was done with a resolution of 2 cm^−1^. Each spectrum was an average of 28 scans. Lastly, analysis of the FTIR spectral data was performed.

### 2.7. Monitoring of Ultrasonic Fields during Nano-ZnO Synthesis Process by Ultrasonic Method

**Figure 1:**
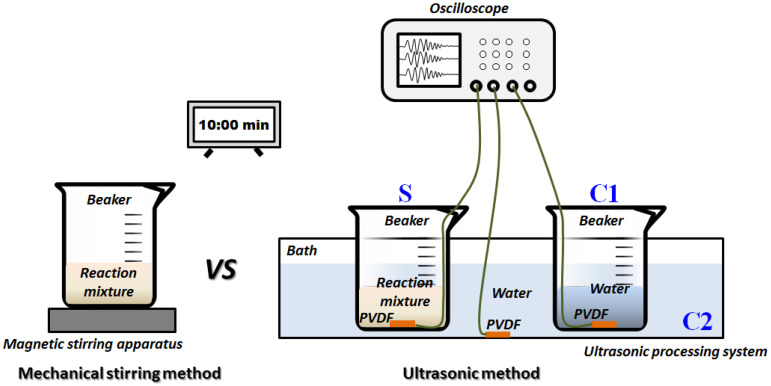
Schematic diagram of monitoring of ultrasonic fields during synthesis process of nano-ZnO.

During the synthesis process of nano-ZnO by the ultrasonic method (Section 2.3), the voltage fluctuations of different ultrasonic fields were measured by a core device called a polyvinylidene fluoride (PVDF) sensor. The effective area of the PVDF sensor was 10 mm × 10 mm, the thickness was 30 μm, and the sensitivity was 2 × 10^−8^ V/Pa. The resistance of 50 Ω in parallel connection was to improve stability of the signal acquisition, and to possibly avoid the distortion. The 1st PVDF sensor was fixed at the bottom of the 100 mL beaker with the reaction mixture, and was used to monitor the ultrasonic field in the reaction mixture, and the group was named **S**. At the same time, the 2nd PVDF sensor was fixed at the bottom of another 100 mL beaker with the same volume (40 mL) of water, and was used to monitor the ultrasonic field in the internal water, and the group was named **C1**. The 3rd PVDF sensor was fixed at the bottom of the bath of the ultrasonic processing system with 2300 mL water, and was used to monitor the ultrasonic field in the external water, and the group was named **C2**. The level of the reaction mixture in **S** and the water in **C1** was lower than the level of the water in **C2**; hence, the liquids in the beakers were affected by the ultrasonic waves. Next, the three PVDF sensors were connected to the corresponding channels of the oscilloscope. Finally, the instantaneous pressure could be transformed into an electrical signal by the PVDF sensor, and recorded and stored via an oscilloscope in the form of voltage signal every 1 min, throughout the whole synthesis time of nano-ZnO of 10 min. The schematic diagram of the synthesis of nano-ZnO and the monitoring of the ultrasonic fields are displayed in Figure 1.

The data of voltage waveform from the ultrasonic fields can be converted to the acoustic pressure; therefore, the space peak temporal peak acoustic intensity, *I_SPTP_*, and the space peak temporal average acoustic intensity, *I_SPTA_*, can be deduced by using Equations (3) and (4) [36].

(3) $$I_{SPTP}=\frac{U_{max}^{2}}{M_{L}^{2}\rho c}$$

where *U_max_* is the maximum instantaneous value of voltage, V; *M_L_* is the sensitivity of PVDF sensor with the value of 2 × 10^−8^, V/Pa; *ρ* is the density of liquids, kg/m^3^, and the density of the reaction mixture is 1.21 × 10^3^ kg/m^3^, and that of water is 1.0 × 10^3^ kg/m^3^; *c* is the acoustic velocity, m/s, and the acoustic velocity in the reaction mixture is 1.904 × 10^3^ m/s, and that in the water is 1.5 × 10^3^ m/s.

(4) $$I_{SPTA}=\frac{F}{M_{L}^{2}\rho c}\int_{t_{1}}^{t_{2}}U_{t}^{2}dt$$

where *F* is the repetition frequency of ultrasonic pulse, Hz; *t*_1_ is the initial time of a single ultrasonic pulse, s; *t*_2_ is the terminal time, s; *U_t_* is the fitting voltage waveform by Origin software. Lastly, the integral was figured out by Matlab software.

## 3. Results

### 3.1. Characterization of Nano-ZnO Micromorphology by AFM

**Figure 2:**
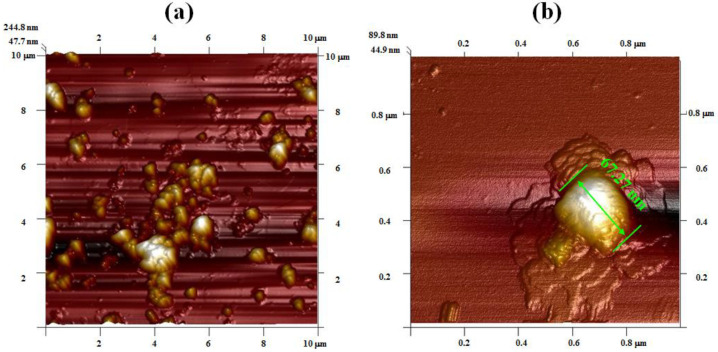

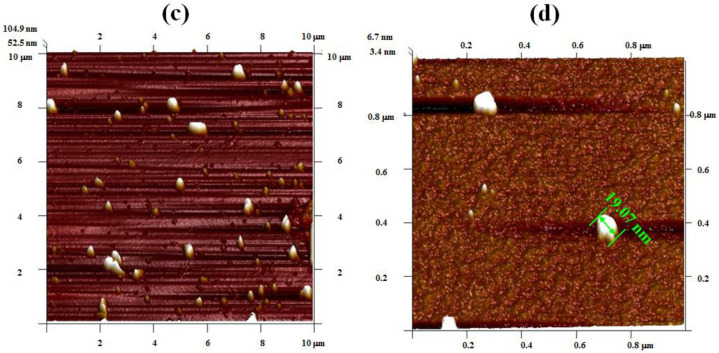
AFM images of nano-ZnO. (**a**,**c**) Surface topography of nano-ZnO by mechanical stirring method and ultrasonic method, respectively; (**b**,**d**) size of nano-ZnO by mechanical stirring method and ultrasonic method, respectively.

The AFM images show the micromorphology of nano-ZnO synthesized by mechanical stirring and ultrasonic methods (Figure 2a–d). The nano-ZnO synthesized by the mechanical stirring method is characterized by uneven distribution and more agglomeration, with an average particle size of 67.3 nm (Figure 2a,b). On the contrary, the distribution of nano-ZnO synthesized by the ultrasonic method is homogeneous and more uniform, and the agglomeration is less. The particle shape is more regular, with an average size of 19.07 nm (Figure 2c,d). As seen from the AFM images, the synthesis of nano-ZnO assisted by ultrasound radiation is obviously superior to that by the mechanical stirring method. The smaller the size of nano-ZnO, the better the antibacterial activity; the nanometer size-effect of nano-ZnO showed high anti-bactericidal activities, and can be used in the postharvest product storage of strawberries [8]. It was considered that the homogeneous dispersion and stability of nano-ZnO in the solution was essential; for example, the adsorption capacity was improved to purify water [2]. The homogeneous nano-ZnO particles obtained by the ultrasonic method can mainly be ascribed to the acoustic cavitation. In more details, the vibration of the acoustic source is generated by an ultrasonic generator, causing the vibration of adjacent liquid molecules, which are then driven during the diffusion process. Micro-streaming is introduced as the implosive collapse of ultrasonic cavitation bubbles; thus, the corresponding shearing actions prevent agglomeration of the particles. Due to a mixing effect of acoustic streaming, intensive perturbed behavior occurs in the local reaction mixture, and homogenization of the reaction system is realized [21]. The high temperature and pressure and the gradient flow induced by the acoustic cavitation enhance the process of mass transfer, and concurrently reduce the surface free energy of nano-ZnO. As a result, the agglomeration of particles is avoided effectively. The collapse of cavitation bubbles produces a tremendous pressure; the shock wave leads to an enhanced tendency towards the fracture of the structure into small-sized nano-ZnO particles. Moreover, the distribution of ultrasonic field prompts the formation of crystal nuclei of nano-ZnO and controls its growth, which provides a good foundation for the synthesis of uniform nano-structure materials. Furthermore, the uniform and fine nano-ZnO corresponds to high surface energy and large specific surface area, so it can be dispersed in the matrix, and promotes the interface bonding and development of food packaging materials with certain antibacterials [6].

The surface roughness of nano-ZnO is listed in Table 1. The surface roughness indicates the change in the degree of surface morphology and aggregation of particles. It is a quantified indicator and represents an average degree of the surface morphology over the surface. It can be found that the surface roughness of nano-ZnO synthesized by the mechanical stirring method is 4–6 times higher than that by the ultrasonic method. The surface roughness is always related to the properties of nano-structure materials. If the surface roughness is higher, the favorable point of strength will be provided for the bacteria through the concave and convex structure. Conversely, when the surface roughness is lower, the probability of bacterial adhesion is reduced, and the antibacterial effect is obvious [5]. It was also considered that the physical conditions of ZnO biological films should go against bacterial adhesion; thus, bacterial proliferation can be inhibited [37]. Hence, more advantageous values of the ultrasonic method will be utilized for the synthesis of antibacterial materials.

### 3.2. Characterization of Nano-ZnO Crystal Structure by XRD

**Figure 3:**
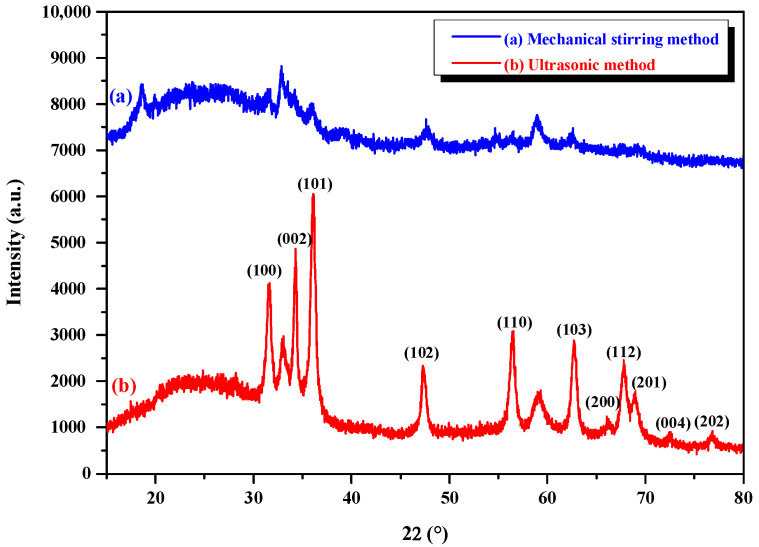
XRD patterns of nano-ZnO synthesized by mechanical stirring and ultrasonic methods.

The XRD patterns of nano-ZnO synthesized by the assist of mechanical stirring and ultrasonic methods are depicted in Figure 3. XRD is an important method to distinguish the crystal structures of materials. The XRD patterns demonstrate that ZnO is crystalline in nature, and the diffraction peaks match well with the standard pattern of the hexagonal wurtzite structure of ZnO (JCPDS card number 36-1451). However, the nano-ZnO synthesized by the mechanical stirring method shows broad-shaped diffraction peaks of lower intensities (Curve a), indicating that the nanostructure has inferior dispersity and crystallinity. On the contrary, the nano-ZnO synthesized by the ultrasonic method is characterized by sharp diffraction peaks of higher intensities (Curve b), indicating that the nanostructure has better, uniform dispersity and wonderful crystallinity. In addition, the intensities of diffraction peaks labeled with the indices of (002) and (100) in Curve b are stronger than those in Curve a. This illustrates that nano-ZnO-preferred growth along the [0001] direction is induced in the ILs under the action of ultrasonic cavitation, with stable thermodynamics for the preparation of food packaging materials [38]. It was also found that polymer chain aggregation was resisted by high numbers of Zn^2+^ ions, and the flow under heating and shearing was enhanced, which is a benefit to the shelf-life extension of meat environmental packaging [9]. Moreover, Zn was proven to be the safe compound for food applications, and is used in the acceptable range for food consumers [10].

Based on the aforementioned analysis, it is found that the crystal growth is controlled effectively in the ultrasonic field, and the strengthened effect on the nucleation and crystallization process is obvious [3]. As the crystal nuclei are generated, the exciting-vibration action can be provided for the growth of temporary crystal nuclei under the intense orientation effect of ultrasonic radiation. Moreover, the crystal surface is denuded and renewed constantly, due to ultrasonic cavitation when the micro-streaming acts on its surface as the collapse of cavitation bubbles occurs. Thus, the crystal growth is accelerated, and the nonuniformity of the growth rate may be restrained along every surface direction of the crystal [39]. Consequently, the shape of the crystal nuclei is regular, integral, and glabrous, and the size distribution of crystal nuclei and crystal products is concentrated, and hence, the coefficient of variation is low. This agrees with the results displayed in Figure 2d and Table 1.

### 3.3. Characterization of Nano-ZnO Structure by FTIR

**Figure 4:**
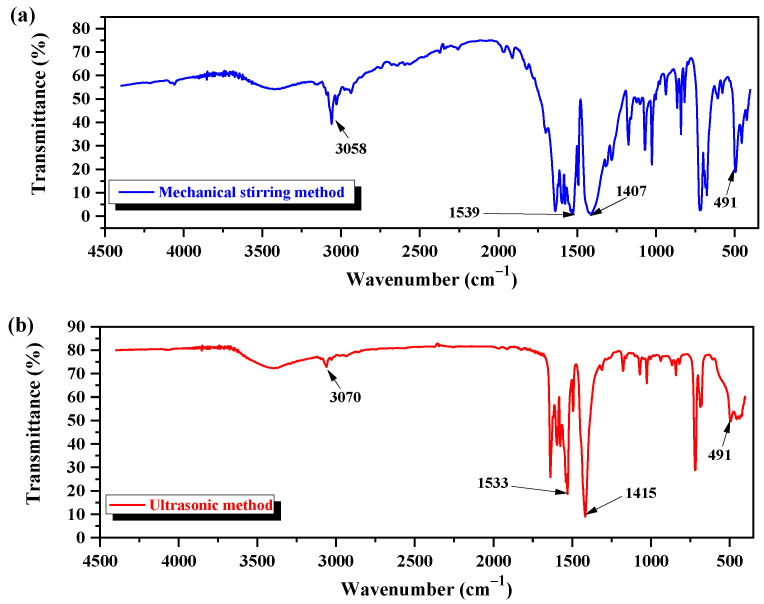
FTIR spectra of nano-ZnO. (**a**) By mechanical stirring method; (**b**) by ultrasonic method.

The FTIR spectral patterns of nano-ZnO synthesized by mechanical stirring and ultrasonic methods are shown in Figure 4a,b. The FTIR spectra of both nano-ZnO are located in the middle infrared band. There are four main factors of the FTIR spectrum: peak position, peak intensity, peak shape, and peak number. The broad peaks located in the wavenumber range of 2700–3300 cm^−1^ correspond to the stretching vibration of the C-H bond, and are found in both nano-ZnO. The absorption peak at 3058 cm^−1^ observed in nano-ZnO synthesized by the mechanical stirring method in Figure 4a is shifted to a higher wavenumber (3070 cm^−1^) after ultrasound-assisted synthesis of nano-ZnO (Figure 4b). In addition, the intensity of the absorption peak at 3058 cm^−1^ is stronger than the intensity of the peak at 3070 cm^−1^. The variations in the position and intensity between the former peaks could be explained by the fact that the mechanical stirring method is a simple mix process of a mild force, and, therefore, the C-H bond can hardly take part in the oxidation reaction. However, the ultrasound-assisted synthesis of nano-ZnO is driven by the acoustic cavitation. The cavitation bubbles collapse produces high temperature and pressure, micro-streaming, and shearing action in the reaction medium; consequently, a series of chemical and electrochemical reactions are induced. In this context, the chemical bond of the water molecule ruptures, the hydroxyl radical ∙OH with strong oxidation capacity is generated, and the C-H bond is promoted to participate in the oxidation reaction [40]. This ultrasonic activity is weakened at the intensity of the absorption peak at 3070 cm^−1^ (Figure 4b). The broad band at 1900–1200 cm^−1^ corresponds to the stretching vibration of the double bond. Two absorption bands at 1539 cm^−1^ and 1407 cm^−1^ are observed in the FTIR spectrum of nano-ZnO by the mechanical stirring method (Figure 4a). The former absorption band is assigned to the asymmetric stretching vibration of carboxylate radical -COO^−^, and the latter band is attributed to symmetric stretching vibration due to Zn (CH_3_COO)_2_·2H_2_O as the precursor. These two absorption bands are broad-shaped with weak intensity (Figure 4a). The absorption bands at 1533 cm^−1^ and 1415 cm^−1^ observed in the FTIR spectrum of nano-ZnO by the ultrasonic method correspond to the carboxylate anion (Figure 4b). The later absorption bands are sharper in shape and have strong peak intensity. There is a relationship between the characteristic peaks and absorption ones, due to the stretching and bending vibration of the hydroxyl group or bridging one on the surface of nano-ZnO. During the ultrasound-assisted process of nano-ZnO, some water molecules are degraded into adsorbent hydroxyl groups under the effect of ultrasonic cavitation. In addition, there exists an interaction between electrochemistry and mechanical force, besides the original carboxylate radical of the precursor. The hydroxyl group at the inorganic interface participates in the reaction, other oxides will be transformed into oxygen-containing carboxylate, and the characteristic bands of the carboxylate appear stronger (Figure 4b). The absorption band at 491 cm^−1^ is the characteristic peak of the Zn-O bond. The differences in the shapes of peaks appearing in the two FTIR spectra (Figure 4a,b) could be the result of the differences in the preparation methods. However, the particle size of nano-ZnO is small, but the percentage of surface atoms is high and, therefore, the corresponding lattice vibrations are different in the context of the infrared radiation. The significant structure of nano-ZnO is conducive to promoting other technologies to prolong the shelf-life of food, and Professor Zhang’s group found that with the extension of storage time, the bagging situation of sterilized Hongsu chicken dishes showed a decreasing trend, and the comprehensive score of products in regards to the shelf-life was within the acceptable range, because of the nano-ZnO-combined low-frequency microwave technology [41].

Some conclusions can be obtained from the above-mentioned analysis. Firstly, the homogenization and dispersity of nano-ZnO are better after ultrasound-assisted synthesis. Secondly, the nucleation and crystallization process can be controlled effectively in the ultrasonic field, and the crystal structure of nano-ZnO is better. Thirdly, the FTIR spectrum of ZnO nanostructure synthesized by the ultrasonic method is different from that by the mechanical stirring method because ultrasonic cavitation generated during the synthesis process is induced by the collapse of cavitation bubbles. Moreover, this cavitation effect mainly appears in the oscillation process of the bubbles with an acoustic signal. To observe the ultrasonic cavitation visually, the on-line monitoring in real-time was utilized during the whole synthesis process of nano-ZnO by the ultrasonic method. The physical mechanism of the ultrasound-assisted synthesis of nano-ZnO is revealed through the voltage fluctuations of the signal.

### 3.4. Analysis of Ultrasonic Fields during Synthesis Process of Nano-ZnO

#### 3.4.1. Monitoring and Analysis of Voltage Waveforms in Different Ultrasonic Fields

The oscillation behavior of cavitation bubbles is not the same in different liquids, and is also different in the same kind of liquid subjected to the restraint from different interfaces. During the ultrasound-assisted synthesis of nano-ZnO, two control groups (**C1** and **C2**) were set to make the mechanism of ultrasonic field clear for **S** (Section 2.7). Voltage waveforms were monitored in real-time in the three groups, and the change process of the ultrasonic fields was researched.

**Figure 5:**
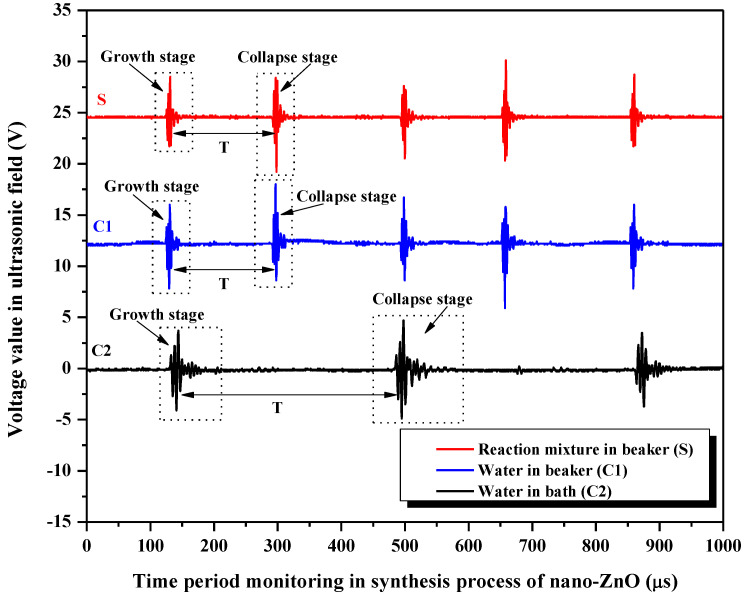
Cyclical fluctuations of voltage waveforms captured from the ultrasonic fields during the synthesis process of nano-ZnO.

Figure 5 shows the cyclical fluctuations of voltage waveforms captured from the ultrasonic fields during the synthesis process of nano-ZnO. The corresponding acoustic signal of ultrasonic cavitation is received through PVDF sensors, and the cyclical oscillation behavior of cavitation bubbles can be represented. The horizontal coordinate represents the time for monitoring, and the vertical coordinate represents the voltage signal of the ultrasonic fields. There are a series of obvious voltage amplitudes for the pulsed ultrasonic fields, so the growth and collapse of cavitation bubbles occur concurrently. The violent collapse of cavitation bubbles results in the production of a more obvious acoustic signal, which can be represented through the voltage waveform. The voltage waveform with smaller amplitude corresponds to the growth of cavitation bubbles, and that with higher amplitude corresponds to the collapse of bubbles, and the time interval between the two adjacent voltage waveforms is the life cycle (T). From the curve of **S**, it can be seen that the effect of ultrasound-assisted synthesis of nano-ZnO is based on the oscillation behavior of cavitation bubbles. In view of the ultrasonic cavitation, the reaction mixture can be mixed by much stronger mechanical force from instantaneous micro-streaming than the conventional mechanical stirring method. Therefore, the preferred growth of the ZnO nanostructure is induced (Section 3.2), and the functional groups are changed (Section 3.3). In addition, the ultrasonic cavitation is also generated in **C1** and **C2**. The cavitation bubbles’ life cycle in **S** is about 166.75 μs, and that in **C1** is about 165.75 μs. The life cycle in **C2** (about 353.75 μs) is two times longer than that in **S** and **C1**. **C1** and **C2** are subjected to the restraint from different interfaces, and obtain different power densities of ultrasonic fields. Higher power density is distributed to **C1** because of its smaller liquid volume; hence, the corresponding acoustic intensity is stronger. In contrast, lower power density is distributed to **C2** because of its larger liquid volume; the corresponding acoustic intensity is weaker. Despite the fewer cavitation nuclei, the lesser liquid volume means a higher power density, and accelerates the oscillation process of cavitation bubbles in **S** and **C1**, and, therefore, the total life cycle is shortened. On the contrary, the larger liquid volume in **C2**, with more cavitation nuclei, results in lower power density, and so, the total life cycle of the cavitation bubbles is prolonged. In our research group, it was also found that there was an important relationship between the life cycle of cavitation bubbles and the ultrasonic effect, and the suitable life cycle corresponded to strong mechanical waves and a significant cavitation effect, which can improve soybean protein content [33] and the soaking efficiency of soybeans [42].

**Figure 6:**
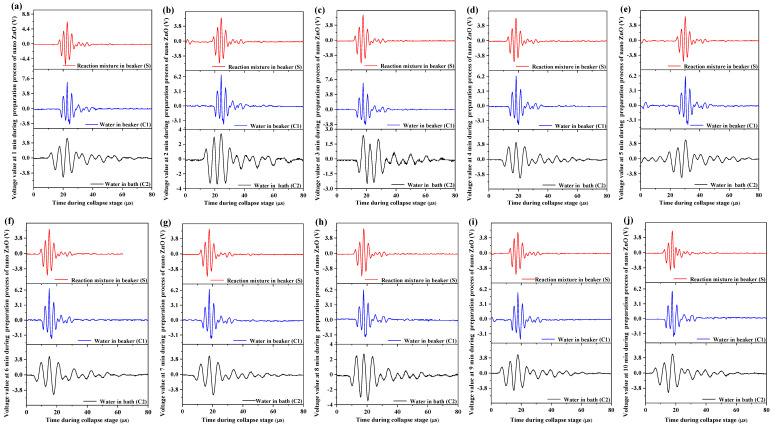
Waveforms at one time of collapse stage acquired every 1 min during ultrasound-assisted synthesis of nano-ZnO. (**a**) 1 min, (**b**) 2 min, (**c**) 3 min, (**d**) 4 min, (**e**) 5 min, (**f**) 6 min, (**g**) 7 min, (**h**) 8 min, (**i**) 9 min, (**j**) 10 min.

In reality, the ultrasonic cavitation effect on the synthesis process of nano-ZnO results from the collapse of bubbles, so the voltage waveforms at the collapse stage should be studied deeply. The research on voltage waveforms has been applied in our earlier study [43], from which it can be gained that voltage waveforms of ultrasonic cavitation fields distinctly visualized the extraction processing of peanut oil and affected the extraction yield. The voltage waveforms at one time of the collapse stage are acquired every 1 min throughout the entire synthesis time of 10 min (Figure 6). Similarly, the voltage waveforms are similar to the symmetrical damped oscillation in S, C1, and C2. At the collapse stage, the maximal bubble radius may be realized after the oscillation process of the shrinking and expanding bubble radius, and the voltage signal is enhanced gradually due to the increasing radiation. When the inside and outside pressure (pressure difference) of cavitation bubbles reaches a certain threshold value, the collapse of bubbles happens. Consequently, high temperature and pressure are induced, and the obvious amplitude of the waveform is obtained, that is, the voltage amplitude. The collapse of cavitation bubbles in the liquid is similar to the explosion, and the bubble can be regarded as an explosion point, and, hence, the scope of the ultrasonic cavitation effect diffuses around in the instant of collapse. However, the influential force of the violent bubbles collapse becomes smaller as time progresses and restores to calmness in the end. The time of the collapse of the cavitation bubble is very short, less than 80 μs (Figure 6). As the collapse of the ultrasonic cavitation bubble is very drastic, the interfaces of every phase in the liquids are affected by the micro-streaming, with a high flow velocity of 100 m/s. The particle size of the aggregates is reduced through the shearing actions, and the materials are homogenized (Section 3.1). At the same time, the nucleation and crystallization process of nano-ZnO is promoted and controlled.

In comparison, the cycle of collapse is almost the same in **S** and **C1**, but is longer in **C2**, which follows the same trend as the life cycle of the bubble, T (Figure 5). Despite the fact that the properties of their liquids are different, the oscillation behaviors of cavitation bubbles in **S** and **C1** can hardly be distinguished accurately because of the small liquid volume used and the short collapse process. Moreover, although the two control systems (**C1** and **C2**) contain water, they are subjected to different restrictive ranges of the interfaces. The corresponding power densities of their ultrasonic fields are different; hence, different effects of the mechanical force on a single water molecule are found in **C1** and **C2** [43]. The far larger water volume in **C2** than that in **C1** leads to smaller ultrasonic power density; accordingly, smaller corresponding voltage amplitude and acoustic intensity are observed in **C2**. The growth and collapse of cavitation bubbles will happen just as the voltage amplitude and the acoustic intensity reach a certain threshold value, so the collapse process of the bubbles slows down in **C2**.

#### 3.4.2. Analysis of the Maximum Voltage Amplitude

**Figure 7:**
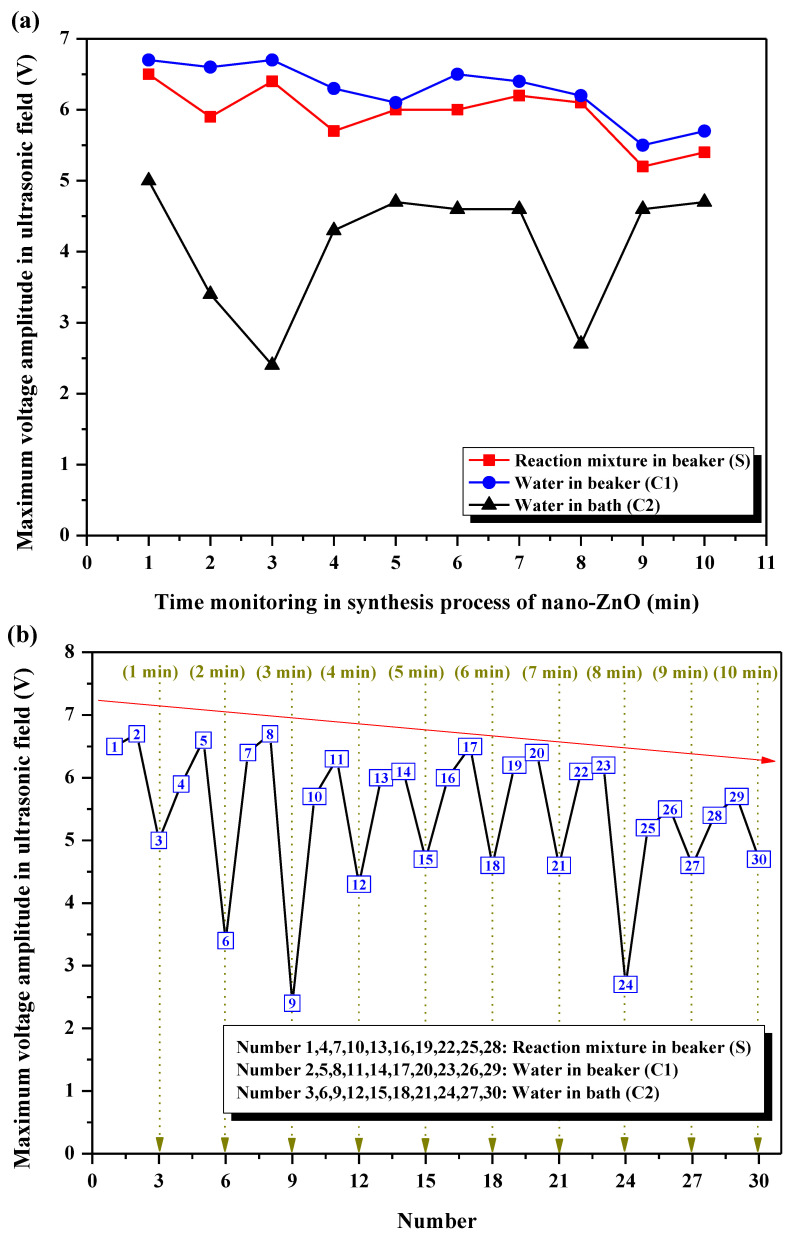
Maximum voltage amplitude during ultrasound-assisted synthesis of nano-ZnO at the collapse stage. (**a**) Respective difference of **S**, **C1**, and **C2**; (**b**) analysis of **S**, **C1**, and **C2** together.

The maximum voltage amplitude at each collapse stage of cavitation bubbles recorded for **S**, **C1**, and **C2** are presented in Figure 7a. **C1** reveals the highest maximum voltage amplitudes, followed by **S**, and then by **C2**. The high maximum voltage amplitudes in **S** are due to the effects of bubble collapse (more than 5 V), resulting in high acoustic intensity and, at the same time, high shock waves. As a result, a developed instantaneous shearing action in the ultrasonic field prevents the formation of nano-ZnO aggregates, and imposes the formation of the nano-sized particles (Section 3.1). The differences in the maximum voltage amplitudes among **S**, **C1**, and **C2** could be explained by the following facts. The reaction liquids in **S** and **C1** are [Bmim][BF_4_] and water, respectively. As is known, water is characterized by a smaller viscosity and larger surface tension compared with ionic liquids. The smaller the viscosity is, or the larger the surface tension is, the stronger the mechanical force of micro-streaming is as the collapse of cavitation bubble occurs [44]. Thus, a stronger acoustic signal can be received by the PVDF sensor with its larger deformation. At the same time, the more obvious the acoustic signal is, the more obvious the electric signal is, and, hence, the higher the maximum voltage amplitude is. Furthermore, the ultrasonic cavitation is generated when the ultrasonic waves propagate in the advantageous liquids. Due to the restraint of different interfaces, the power density of the ultrasonic field in **C1** is larger than that in **C2**, and stronger acoustic intensity and impact force are obtained. As a result, the more obvious voltage signal is acquired by the PVDF sensor, and the maximum voltage amplitude in **C1** is higher than that in **C2**.

The maximum voltage amplitudes in **S**, **C1**, and **C2** are compared and analyzed together during the whole ultrasound-assisted synthesis process of nano-ZnO (Figure 7b), and the regularity among them can be obtained. The maximum voltage amplitude in **S** is higher than that in **C2** at one minute during the synthesis process. The research considers that the effect of ultrasonic cavitation is more obvious when the reaction mixture is in a direct contact with the ultrasonic field, such as in probe-type sonicators. Some ultrasonic energy can hardly be transferred through the liquid medium to the reaction mixture in the vessel, but can be introduced directly into the system [20]. Moreover, the general trend of the maximum voltage amplitudes decreases. Some cavitation bubbles may disappear after the oscillation process, which is also the defoaming principle of ultrasound. Then, no more bubbles will participate in the oscillation process, the cavitation intensity and the corresponding signal will get weaker, and the maximum voltage amplitude will also decrease.

#### 3.4.3. Analysis of Space Peak Temporal Peak Acoustic Intensity and Space Peak Temporal Average Acoustic Intensity

In biological ultrasonic diagnosis, with the problem of biosafety dose, ultrasonic acoustic intensity has been paid more and more attention because ultrasonic acoustic intensity has an important impact on the health and safety of organisms, and reasonable ultrasonic acoustic intensity can avoid the generation of bad biological effects. Space peak temporal peak acoustic intensity, *I_SPTP_*, and space peak temporal average acoustic intensity, *I_SPTA_*, are two kinds of ultrasonic acoustic intensity closely related to the ultrasonic effect of organisms [36]. Table 2 shows *I_SPTP_* in **S**, **C1**, and **C2** during ultrasound-assisted synthesis of nano-ZnO. The values of the maximum voltage amplitude (Figure 7) are plugged into Equation (3), resulting in *I_SPTP_*. The value of *I_SPTP_* in **S** is lower in comparison with the value in **C1**; the lowest *I_SPTP_* value is shown by **C2**. As seen, the results of *I_SPTP_* in **S**, **C1**, and **C2** are in accord with the records of the maximum voltage amplitude (Section 3.4.2). For *I_SPTP_* in **S**, the ultrasonic cavitation is enough to be induced, the synthesis process is promoted, and nano-ZnO with excellent properties can be gained. This interaction strength cannot be realized by the conventional mechanical stirring method. The liquid volume in **S** is equal to that in **C1**, but the surface tension of the liquid in **S** is smaller, and it is hard to split the bubble wall. Furthermore, the propagation velocity of ultrasonic waves is faster in **S**, and the action time gets so short that the collapse of cavitation bubbles is not generated while the ultrasonic waves propagate out of the reaction mixture. Comparatively speaking, there is enough action time for the ultrasonic waves to interact with the liquid in **C1**, and the drastic oscillation behaviors of cavitation bubbles are induced. As a result, an obvious cavitation effect with stronger *I_SPTP_* is observed. In addition, the power density of the ultrasonic field is smaller in **C2**; the driving force is so small that the oscillation process cannot proceed to the collapse stage; and *I_SPTP_* is weaker in the local region. In fact, the ultrasonic cavitation intensity can be represented by *I_SPTP_*, and the prediction of the cavitation effect will be completed through the monitoring of *I_SPTP_* [45].

The curve fitting of the voltage waveform (Figure 6) is calculated by Origin software, and the functional relationship between voltage waveform, *U_t_*, and time, *t*, can be obtained and expressed as:

(5) $$U_{t}=-0.25+6.77e^{\frac{-{\left(t-30.23\right)}^{2}}{0.19}}$$

Equation (5) is plugged into Equation (4), and the space peak temporal average acoustic intensity, *I_SPTA_*, is gained (Table 3). *I_SPTA_* in **S** is high enough that the synthesis of nano-ZnO can be completed successfully and the corresponding property is fine. In comparison, *I_SPTA_* in **C1** is stronger due to the different properties between ILs and water. In addition, *I_SPTA_* in **C2** is the strongest. Although *I_SPTP_* in the local region of **C2** is the weakest (Table 2), more cavitation nuclei occur due to the largest liquid volume, and the occurrence probability of the cavitation effect is the highest with the summation of all the cavitation nuclei in the local region.

## 4. Conclusions

In this study, the micromorphology and microstructure of nano-ZnO obtained by the ultrasonic method were compared with that by the mechanical stirring method. The on-line monitoring of different ultrasonic fields was studied in real-time during ultrasound-assisted synthesis of nano-ZnO (**S**). Two control groups (water in beaker (**C1**) and water in bath (**C2**)) were set, and the corresponding mechanism was revealed emphatically. Some important concluding remarks are stated below.

(1) The AFM analysis indicated that nano-ZnO synthesized by the ultrasonic method was characterized by a smaller structure and surface roughness, and better dispersity, compared with that by the mechanical stirring method. According to the XRD analysis, the nucleation and crystallization process was controlling effectively in the ultrasonic field, resulting in nano-ZnO with a better crystal structure. FTIR spectral analysis proved the occurrence of some differences in the ZnO nano-structure by ultrasonic and mechanical stirring methods.
(2) Under the ultrasonic field, the reaction mixture during the synthesis of nano-ZnO **S** and the control group **C1** showed shorter life cycles of cavitation bubble oscillation compared with **C2**. The cycle of collapse stage in **S** and **C1** was almost the same, but it was longer in **C2**. The maximum voltage amplitudes in **C1** were generally higher than that in **S**, and were the lowest in **C2**. A general trend in the maximum voltage amplitudes in **S**, **C1**, and **C2** was to decrease as the action time prolonged. *I_SPTP_* in **S** was lower than that in **C1**, and *I_SPTP_* in **C2** was the lowest. *I_SPTA_* in **C1** was stronger than that in **S** due to differences in the properties between ILs and water. *I_SPTA_* in **C2** was the strongest.

In the future, nano-ZnO prepared by direct ultrasound-assisted preparation will also have a wide range of application values in the food areas due to its advantages of large specific surface area and good dispersion, such as high-performance food packaging preparation, adsorption and purification of water resources, food-quality testing sensors, and in the anticorrosion and antibacterial properties of food-machinery stainless steel.

## Figures and Tables

**Table 1:** The surface roughness of nano-ZnO by mechanical stirring and ultrasonic methods.

	Surface Roughness (nm)	
	*R_a_*	*R_q_*
Mechanical stirring method	25.6	43.1
Ultrasonic method	4.2	10.5

**Table 2:** Space peak temporal peak acoustic intensity *I_SPTP_* in **S**, **C1**, and **C2** during ultrasound-assisted synthesis of nano-ZnO.

Synthesis Time	Space Peak Temporal Peak Acoustic Intensity *I_SPTP_* (W/m^2^) × 10^10^		
	Reaction Mixture (S)	Water in Beaker (C1)	Water in Bath (C2)
1 min	4.6	7.5	4.2
2 min	3.8	7.3	1.9
3 min	4.4	7.5	1.0
4 min	3.5	6.6	3.1
5 min	3.9	6.2	3.7
6 min	3.9	7.0	3.5
7 min	4.2	6.8	3.5
8 min	4.0	6.4	1.2
9 min	2.9	5.0	3.5
10 min	3.2	5.4	3.7

**Table 3:** Space peak temporal average acoustic intensity *I_SPTA_* in **S**, **C1**, and **C2** during ultrasound-assisted synthesis of nano-ZnO.

Liquid	Space Peak Temporal Average Acoustic Intensity *I_SPTA_* (W/m^2^) × 10^7^
Reaction mixture (**S**)	6.4
Water in beaker (**C1**)	9.9
Water in bath (**C2**)	33.3

## Data Availability

The datasets generated for this study are available on request to the corresponding author.
